# Patients’ Utilization and Perception of an Artificial Intelligence–Based Symptom Assessment and Advice Technology in a British Primary Care Waiting Room: Exploratory Pilot Study

**DOI:** 10.2196/19713

**Published:** 2020-07-10

**Authors:** Stephen Miller, Stephen Gilbert, Vishaal Virani, Paul Wicks

**Affiliations:** 1 NHS Paxton Green Group Practice London United Kingdom; 2 Ada Health GmbH Berlin Germany

**Keywords:** human-centered design, innovative, health care apps, eHealth, symptom checker, primary care, general practice, app, usability, acceptability, utility

## Abstract

**Background:**

When someone needs to know whether and when to seek medical attention, there are a range of options to consider. Each will have consequences for the individual (primarily considering trust, convenience, usefulness, and opportunity costs) and for the wider health system (affecting clinical throughput, cost, and system efficiency). Digital symptom assessment technologies that leverage artificial intelligence may help patients navigate to the right type of care with the correct degree of urgency. However, a recent review highlighted a gap in the literature on the real-world usability of these technologies.

**Objective:**

We sought to explore the usability, acceptability, and utility of one such symptom assessment technology, Ada, in a primary care setting.

**Methods:**

Patients with a new complaint attending a primary care clinic in South London were invited to use a custom version of the Ada symptom assessment mobile app. This exploratory pilot study was conducted between November 2017 and January 2018 in a practice with 20,000 registered patients. Participants were asked to complete an Ada self-assessment about their presenting complaint on a study smartphone, with assistance provided if required. Perceptions on the app and its utility were collected through a self-completed study questionnaire following completion of the Ada self-assessment.

**Results:**

Over a 3-month period, 523 patients participated. Most were female (n=325, 62.1%), mean age 39.79 years (SD 17.7 years), with a larger proportion (413/506, 81.6%) of working-age individuals (aged 15-64) than the general population (66.0%). Participants rated Ada’s ease of use highly, with most (511/522, 97.8%) reporting it was very or quite easy. Most would use Ada again (443/503, 88.1%) and agreed they would recommend it to a friend or relative (444/520, 85.3%). We identified a number of age-related trends among respondents, with a directional trend for more young respondents to report Ada had provided helpful advice (50/54, 93%, 18-24-year olds reported helpful) than older respondents (19/32, 59%, adults aged 70+ reported helpful). We found no sex differences on any of the usability questions fielded. While most respondents reported that using the symptom checker would not have made a difference in their care-seeking behavior (425/494, 86.0%), a sizable minority (63/494, 12.8%) reported they would have used lower-intensity care such as self-care, pharmacy, or delaying their appointment. The proportion was higher for patients aged 18-24 (11/50, 22%) than aged 70+ (0/28, 0%).

**Conclusions:**

In this exploratory pilot study, the digital symptom checker was rated as highly usable and acceptable by patients in a primary care setting. Further research is needed to confirm whether the app might appropriately direct patients to timely care, and understand how this might save resources for the health system. More work is also needed to ensure the benefits accrue equally to older age groups.

## Introduction

### Background

When a person experiences a new medical symptom, there is an ever-expanding menu of health care–seeking options available. The option they choose may be influenced by factors such as age, sex, the nature of the complaint, chronic ill health, trust in their physician, socioeconomic factors [1], and where applicable, out-of-pocket costs [2]. Within the traditional UK medical system, they might seek care from a hospital emergency department, general practitioner (GP), telephone triage service (eg, 111 in the UK), pharmacist, or urgent treatment center [3]. More recently, internet-enabled options have emerged such as using a search engine to look up symptoms (Dr Google), high-quality online resources such as NHS Choices [4], symptom checkers [5,6], telehealth consultations by phone or videocall [7], *Minute Clinics* [8] that can be booked via smartphone, and peer-to-peer networking [9]. Some two-thirds of patients have searched their symptoms online before a doctor visit [10], with risks of inappropriate information and a lack of appropriate triage for urgent cases [5].

Against the background of an aging population, high burden of chronic conditions, growing consultation rates, and lengthening clinical visits, the overall workload on primary care [11] and emergency medicine [12] is increasing substantially [11]. Accordingly, supply-orientated improvements to traditional processes such as diversion of nonurgent patients [13], nurse triage, fast-tracking [12], and telephone triage [14] seek to more optimally use professional resources. On the demand side, public health campaigns admonish patients via marketing campaigns with blunt messages such as *Don’t go to A&E*. However, in a chronically under-resourced system, making relatively minor adjustments will yield relatively small results [13], and applying a broad approach to dissuading use of medical resources may have unintended negative consequences; most people cannot adequately distinguish between problems that are *urgent*, *emergency*, and *routine care* [15]. While there is much excitement about the potential for video consultations and the UK National Health Service (NHS) GP contract even states “every patient will have the right to online and video consultation by April 2021,” the accumulated experience has been that health IT solutions within the NHS tend to suffer “non adoption, abandonment, and challenges to scale-up, spread, and sustainability” [16,17].

One potentially transformative and more scalable approach to these challenges is digital symptom checkers [5]. Put simply, a patient enters the symptoms they are experiencing in a question-and-answer *chat* format, and receives suggestions as to what the problem might be (diagnostic possibilities), the level of care that would be appropriate (triage), and often the level of urgency with which action should be taken. These software tools rely variously on a digitized body of medical knowledge, decision trees, predictive algorithms, Bayesian inference, and testing against representative case sets to provide accurate advice. Examples include tools developed by health providers such as the Mayo Clinic or NHS as well as private companies. The potential benefits include escalation of urgent cases to appropriate care, the diversion of nonurgent cases to self-care, the deterrence of antibiotic overprescribing, reducing physician burden, less need for telephone triage services [4], saving money for the health system, and saving the patient’s money and time (an average of 3 hours per visit) [5,18].

Patients seem ready to embrace such approaches given the preponderance of technology in their daily lives [19]. A recent survey of over 1000 London residents conducted by Healthwatch Enfield [20] suggested that most patients (63%) would welcome use of a trusted symptom checker, though there were much higher degrees of willingness reported by those under the age of 40 (71%-74% agreed) than over the age of 70 (just 34% agreed). Among the reasons why those surveyed would *not* want to use a symptom checker, concerns were raised over misdiagnosis, health anxiety, digital illiteracy, ease of use, and wanting to see a doctor or nurse face-to-face. Although similar rates of interest were expressed for the use of video consultations (eg, Skype) or email, these would have much higher burdens on professional time than fully digital symptom checkers. This survey has been influential in UK health policy circles, receiving press attention and prompting responses from NHS England and NHSX, a UK government policy unit with responsibility for developing best practice and national policy for technology in health [21].

### Aim

A recent review of patient-facing digital symptom checkers proposed a series of next steps that should be undertaken by the field to evaluate such tools [22]. In this study we used one of the proposed approaches, that is, “Early observational studies in clinical settings” to “test symptom checkers in a safe, observational manner, where patients continue to receive standard care.” We sought to ascertain the usability, acceptability, and utility of one such symptom assessment technology, Ada, in a primary care setting. Our aim was to assess the potential to more effectively meet patient needs and to consider how the use of similar technology at home might improve patient flow in a busy primary care setting. In response to the Healthwatch Enfield report finding a significant factor of age in driving acceptability of symptom checkers, we explored this issue as a secondary question of interest.

## Methods

### Recruitment

Potential participants were initially informed about the Ada study by the clinic receptionist as they were checking in. These potential participants were then approached by an Ada member of staff and asked if they would be interested in testing a new technology, on the understanding that there would be no change to their usual care, that participation was entirely voluntary, and that there would be no compensation for taking part. Potential participants were excluded if they were attending for a nonclinical reason (eg, requesting a doctor’s letter), or if they were attending for a routine chronic disease follow-up appointment (without acute symptoms). If they agreed, participants were given a study smartphone preloaded with a special test version of Ada, completed an assessment, and handed the smartphone back to the research team. A total of 3 study smartphones were in use simultaneously. The research team then asked each participant to complete a paper questionnaire to gather feedback. They then attended their doctor consultation as normal.

### Measures

Participants were asked to complete a paper questionnaire including their full name, date of birth, sex, and Likert-scale multiple choice questions on how likely they would be to recommend Ada, their ease of use, whether Ada provided helpful advice, whether they would use it again, and whether using Ada changed a decision about what to do. A copy of the questionnaire is provided in Multimedia Appendix 1.

### Statistical Analysis

As a descriptive usability and acceptability study, we had no falsifiable hypotheses and so did not undertake a formal power analysis. The sample gathered was based on a convenience sample for the resources available; 2 full-time medical students embedded within the clinic for 5 weeks. Missing data were described per analysis and participants were not excluded for missing data. For comparison with a prior survey, the Healthwatch Enfield report [20], user age was recategorized into the same age groups used in that study, <17 years, 18-24, 25-39, 40-54, 55-69, and 70+. Because data from the <17-year age group were not reported by Healthwatch Enfield [20], they were excluded from usability analysis. A Student *t* test was used for comparison of two group means in normally distributed continuous data. A chi-square test was used to compare nonparametrically distributed or categorical variable differences or both, with statistical significance set at *P*<.05, two-tailed. Statistical analyses were conducted in SPSS version 21 (IBM).

### Ethics

Ethical standards associated with product testing and usability research were applied to this research. To understand the relevant ethical guidelines in the UK, we employed the NHS Health Research Authority decision tool [23], which confirmed this study would not be considered research by the NHS because the study participants were not randomized, did not require a change in standard care, and were not intended to provide generalizable findings outside the setting of interest. All data were securely collected by Ada in a manner compliant with ISO27001 (quality standard for information security). In addition, Ada has a Class I medical device CE mark, is EU General Data Protection Regulation (GDPR) compliant, and is certified by “Bundesverband der Internetmedizin,” the German Federal Association of Internet Medicine.

### Design and Setting

The observational study was conducted between November 2017 and January 2018 at Paxton Green Group Practice, a large primary care clinic in the South London borough of Lambeth with 11 working GPs caring for around 20,000 registered patients. The team is supported by 6 practice nurses, a primary care assistant practitioner, a clinical pharmacist, and an associate physician, alongside 19 administrative and reception staff. Relative to national estimates in the UK, the practice’s population skews younger (aged 25-40 years), having a higher-than-average degree of income deprivation, and with a higher-than-average proportion of black and ethnic minority groups (59% white vs UK population average of 80%) [24], with about 1 in 4 patients identifying as black. A daily *Walk & Wait Clinic* is available each morning for patients without an appointment, from which participants in this study were drawn.

### Description of the Ada Symptom Assessment Tool

**Figure 1:**
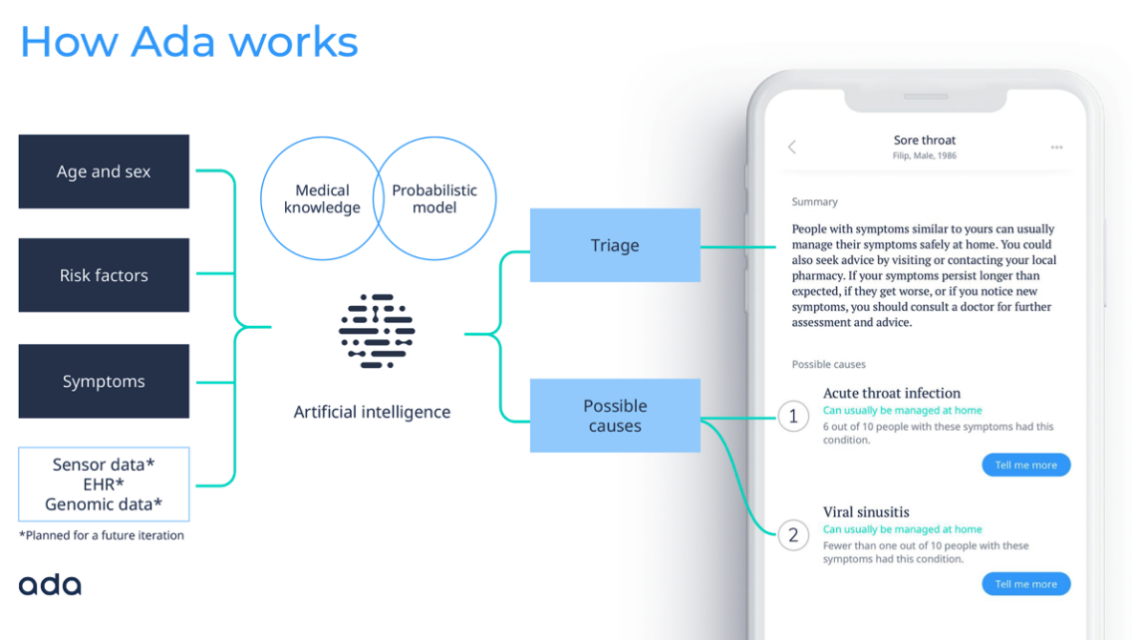
Conceptual overview and screenshot of the Ada symptom checker. EHR: electronic health record.

The basic principles of the Ada medical intelligence are as follows: In the assessment, the user inputs basic health information (eg, age, sex, smoking status, diabetes status), and is then asked for their most troubling current symptoms (presenting complaint). The user is then asked a series of questions by the app, with each question asked being dynamically chosen by Ada’s reasoning engine based on the probabilistically determined optimal question. This question is determined by the reasoning engine, based on all previously supplied basic health information and symptoms. The reasoning engine has been designed to ask a balanced number of questions that allow reasonable identification of conditions from medical history without being overly burdensome to complete (Figure 1).

The reasoning engine infers disease probability estimations based on a representation of medical knowledge. The medical knowledge base is used to define a Bayesian network, on which approximate inference is carried out, and following which information-theoretical methods are used to decide which questions to ask to the user. The knowledge base was built and reviewed by medical doctors in a curated process of knowledge integration from medical literature. It is being expanded continuously following this standardized process. It consists of disease models of all common conditions and several hundred rare diseases, including their corresponding symptoms and clinical findings. The disease models and their related symptoms are added to the knowledge base and modeled according to evidence from peer-reviewed medical literature. Symptoms/clinical findings can be further refined with additional attributes, for example, intensity or temporality and epidemiological data are used to derive the prior probabilities of diseases to allow for correct disease probability estimations. Ada’s medical intelligence (meaning the combination of Ada’s reasoning engine and medical knowledge) is continually validated against a set of several thousand internal test cases, which comprise diseases from different medical specialties and include both common and rare diseases. The set includes cases based on medical literature (eg, published case reports) as well as typical clinical case scenarios that reflect different levels of diagnostic certainty. A team of Ada medical doctors constantly reviews the system’s inherent medical knowledge based on these quality assurance measures. Ada’s medical intelligence is further verified on a continual basis through a second process, in which a verification tool is used to test each update of Ada’s medical intelligence, using hundreds of cases written by external doctors. These cases are kept confidential from the Ada medical doctors who curate the medical knowledge base and the set of cases is regularly updated.

At Ada, usability engineering is directly integrated in the product development process. The usability process and respective activities heavily overlap with general design and user research activities, yet emphasize the importance of documentation and transparency of product decisions. At the beginning of the product development process, generative user research is conducted (eg, user interviews, shadowing, expert interviews) to gain a better understanding of the user and potential opportunities. Insights generated from this phase are passed on to design, where initial concepts, based on user requirements, are crafted. These concepts are often made tangible via prototypes which range from low to high fidelity, so that they can be evaluated with representative end users. Nonetheless, other methods such as heuristic evaluations or cognitive walk throughs are used to gather feedback on the general usability and user experience of the interface. Findings such as use errors or usability problems are then fed back into the next design iteration until a suitable solution has been found. This evaluative work is usually referred to as formative evaluations. They take place throughout the iterative product development until the product reaches its final state to control for risk and ensure safety by design. Prior to release, a summative evaluation (ie, a final evaluation of the product) is conducted to ensure the product is effective and safe to use. Furthermore, after product release, user feedback is collected via surveys, contextual interviews, and large-scale research studies, which is part of the postmarket monitoring activities and can initiate design iterations to improve user experience, usability, and safety of the product. If usability problems or areas of potential usability improvement are identified in the postmarket phase, then design improvements are introduced using the same process as described above.

## Results

### User Statistics

Over a 3-month period, 523 patients completed an Ada assessment and the questionnaire. Although data on nonconsenting patients were not gathered, we estimate that around two-thirds of those approached agreed to participate. Most participants were female (Table 1, n=325, 62.1%), with about one-third male (n=185, 35.3%) and 13 cases with no sex reported. Relative to 2011 UK Census data, and the practice’s own data for all registered patients, this represents a higher proportion of females, although females are known to use health care services more frequently [1].

**Figure 2:**
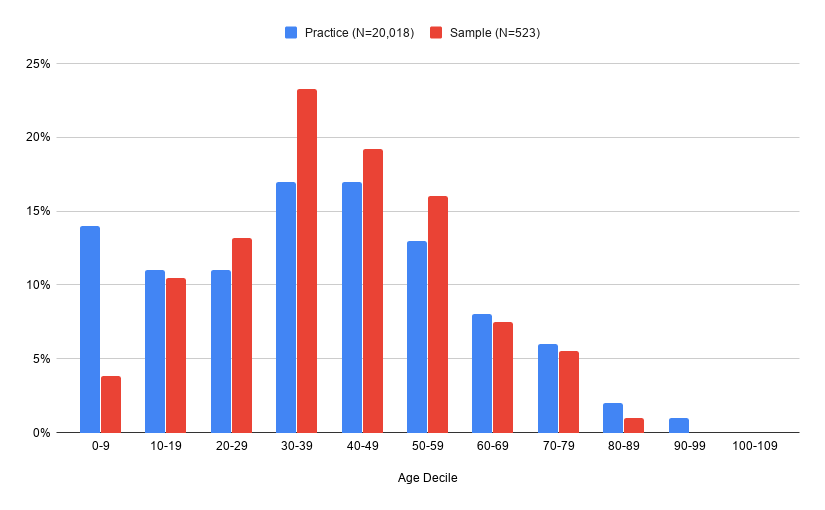
Age distribution of registered patients at the Paxton Green practice compared with sample respondents.

Mean age of patients was 39.79 years (SD 17.7 years), with age data missing for 17 participants (3.3%). Relative to 2011 UK Census *broad age group* data, this population had a larger proportion (81.6%) of working-age individuals (aged 15-64) than the general population (66%), with smaller proportions of children (aged 0-14, 7.9% vs 18% nationally) and smaller proportions of older people (aged 65+, 10.5% vs 16% nationally). There were no significant differences in mean age between males (39.05 years, SD 19.06) and females (40.27 years, SD 17.00) using the Student *t* test (t_501_=.739, *P*=.460). Relative to the practice’s registered population, the sample included fewer parents reporting on behalf of children and more middle-aged adults (Figure 2).

**Table 1:** Participant sex distribution compared with practice population.

Sex	Sample, n (%)^a^	Practice, n (%)^b^
Female	325 (62.1)	10,331 (51.61)
Male	185 (35.3)	9687 (48.39)
Not reported	13 (2.4)	0 (0)

^a^N=523.

^b^N=20,018.

### Usability and Acceptance Testing

Overall, participants rated ease of use highly, with most participants (348/522, 66.7%) reporting it was *very easy* to use Ada; most of the remaining participants reported *quite easy* (163/522, 31.2%), with just 11 reporting issues (9/522, 1.7%, *quite difficult*; 2/522, 0.4%, *very difficult*; and with 1 participant missing data). As shown in Table 2, relative to the Healthwatch Enfield study, we saw a much higher degree of acceptance from actual users who had interacted with Ada than from (an admittedly different) group of survey respondents being asked how likely they thought they would be to use a (unspecified) symptom checker.

While we had no preplanned hypotheses to test statistically, inspection of the means suggests that there is a trend for higher levels of enthusiasm, utility, willingness to use again, potential impact on clinical decisions, and potential diversion away from clinic by age group. For example, while 22% (11/50) of those aged 18-24 suggested that using Ada would have changed a decision had they used it before attending the GP, no patients over the age of 70 (0/28, 0%) agreed with this statement (though numbers were small, 28/427 respondents or 6.6% of the sample). Nonparametric chi-square testing found no sex differences on any of the usability metrics described in Table 2 (analysis not shown).

**Table 2:** Usability and acceptance responses stratified by Healthwatch Enfield [20] respondent age categories.^a^

Age category	Healthwatch Enfield “would use a symptom checker before seeking advice from GP” (N=1071)^b^	Extremely Likely/Likely to recommend Ada to a friend or relative (N=447)	Very/Quite easy to use Ada (N=450)	Yes, Ada Provided Helpful Advice (N=437)	Yes, Would Use Ada Again (N=433)	Yes, Using Ada Changed a Decision (N=427)	Yes, Would Still Have Come to Clinic if Had Used Ada Before (N=443)
18-24, n/N (%)	74	50/54 (92.60)	54/54 (100)	49/53 (92.45)	50/54 (92.59)	11/50 (22.00)	51/53 (96.23)
25-39, n/N (%)	71	125/147 (85.03)	146/147 (99.32)	116/145 (80.00)	129/145 (88.97)	17/140 (12.14)	132/145 (91.03)
40-54, n/N (%)	69	121/141 (85.82)	137/143 (95.80)	108/138 (78.26)	120/133 (90.23)	19/137 (13.87)	125/140 (89.29)
55-69, n/N (%)	51	64/72 (88.89)	72/73 (98.63)	53/69 (76.81)	59/70 (84.29)	11/72 (15.28)	66/72 (91.67)
70+, n/N (%)	34	25/33 (75.76)	32/33 (96.97)	19/32 (59.38)	22/31 (70.97)	0/28 (0.00)	32/33 (96.97)

^a^N values vary due to missing data; n=17 did not provide age and n=56 participants under the age of 17 were excluded from this comparison.

^b^Only percentage is reported due to missing n/N value.

### Urgency Advice Levels and Redirection

One aim of a digital symptom assessment tool is to give appropriate advice and, where appropriate, to encourage self-care (eg, self-limiting illnesses such as upper respiratory infections). Participants were asked to self-report whether using the Ada assessment would have changed their decisions about what to do next. Overall, most respondents (425/494, 86.0%) said they would not have changed their decision, with other responses shown in Table 3.

**Table 3:** Self-reported predicted change in care navigation as a result of using a symptom checker.

Did using Ada change your decision about what to do next?	n (%)^a,b^
No	425 (86.0)
Yes—Changed my mind from wanting to see a GP^c^ to self-care at home	23 (4.6)
Yes—Changed my mind from wanting to see a GP to visiting the pharmacy	20 (4.0)
Yes—Changed my mind from wanting a same-day appointment to delaying my appointment for a few days	20 (4.0)
Yes—Changed my mind from wanting to see a GP to visiting A&E^d^	6 (1.2)

^a^Missing data: 29.

^b^Total valid entries: 494.

^c^GP: general practitioner.

^d^A&E: accident & emergency.

## Discussion

### Principal Results

In this real-world usability study, participants in a South London primary care setting endorsed Ada’s ease of use, with the majority saying they would use Ada again. These data from people given the opportunity to use a real product contrast with the Healthwatch Enfield report survey collected in a similar time range in the same city where respondents asked by survey whether they would, in theory, be willing to use a briefly described symptom checker were less enthusiastic, particularly those in older age groups [20].

Given the product’s intent of providing improved access to health care to everyone, it was reassuring to find no sex differences in perceived usability or utility of the symptom checker app. However, we did find age differences on several key factors including willingness to use again, perceived usefulness, and likelihood of changing a health decision. Prior research in the field has identified age-related differences in willingness to use technology [20], but this is also confounded by the nature of the health problems presented by different age groups. For example, a number of apps have reported a much younger user base than the general population, and younger users may also reflect more engaged users.

Although speculative, the fact that older people found the app just as easy to use but reported less engagement might suggest that the issue is not one of usability or familiarity with technology. Rather, future research could explore whether older potential users might have more interest in face-to-face interaction with a clinician, want to discuss chronic conditions or issues of multimorbidity, or that, having had more experience with the health system, they might see potential risks in a digital approach that younger people may not perceive.

### Limitations

As a small feasibility study, our approach had a number of limitations which we will seek to address with hypothesis-driven research in the near future. Asking patients already in a GP’s waiting room what they *might have done* in a questionnaire may have poor predictive validity compared with other markers such as their prior behavior [25]. Unmeasured factors in this study such as the quality of a patient’s relationship with the GP have been shown to be an important driver of health-seeking behavior and should be taken into account [26]. While most respondents said using the symptom checker would not have changed their decision to see the GP, it is worth noting that at the point of enrollment they were on the cusp of seeing their doctor face-to-face, and were therefore quite committed to their current path. Our sample contained a higher proportion of females than either the practice’s data suggest are registered or the UK census data; this may be explained by females being more frequent users of health care services [1]. Future studies should study real-world patient behavior before they have a clinic appointment booked.

### Comparison With Prior Work: Usability

This study suggested a high degree of usability, with nearly all respondents (511/522, 97.8%) reporting a high degree of ease of use. Similarly, an independent study by an external academic group unrelated to Ada sought to understand the applicability of a multidimensional short form *User Engagement Scale* [27] in mobile health apps, using the Ada symptom checker as an example [28]. In a convenience sample of 73 German-speaking Swiss participants (49% female; mean age 39 years, SD 15.4 years; range 18-73), they reported ratings were high for *perceived usability* and *aesthetic appeal* [28]. Studies of other symptom checkers also report a high degree of perceived utility. In a convenience sample of 304 US users of the Isabel symptom checker, 90.1% (274/304) agreed or strongly agreed that it gave them useful information, and a similar proportion said they would use the tool again [29].

### Comparison With Prior Work: Redirection

In terms of reducing the burden on primary care, some 12.8% (63/494) of respondents in this study predicted that they would have used a less urgent care option such as a pharmacist or self-care had they used Ada before visiting the doctor. It remains to be seen how many patients would actually follow advice on where to go next, but in the survey of US Isabel symptom checker users, about half (14/26, 54%) of those advised to go to the emergency department reported that they did so [29]. Another recent paper reported broadly similar findings from over 150,000 encounters with the Buoy Health symptom checker: 18.8% of patients who had planned to visit primary care reduced the urgency of care they would seek, and 2.6% increased the urgency of their intended level of care [30]. The differences in findings between the studies are not large, and likely primarily reflect the major design difference between the studies: our study explored those patients who have already chosen to attend the primary care practice, whereas the Buoy study explored intentions expressed at home. Both approaches have advantages and disadvantages: this study excluded those patients who would later change their mind about attendance after app use, whereas patient intention may have changed after being recorded in the Buoy study, even without changed symptoms. Our study explores a patient population who made a proactive decision to attend the surgery: likely a population with more severe symptoms. Other likely less significant reasons for differences in results between the two studies may be associated with cultural differences (UK vs USA), differences in the platform (mobile phone vs web based), and differences in the presentation of advice levels between the two symptom assessment apps.

### Iterative Product Improvements in Response to User Feedback

One limitation of the Ada version used in this study was difficulty interpreting many of the phrases that patients used to express their initial symptoms as free text. We have sought to address this poststudy by developing a more sophisticated approach to recognizing the free text phrases patients are using to describe their symptoms. This approach leverages machine learning, which is applied if the user query does not match any results in an internal library of recognized terms and phrases. The machine learning approach then suggests entities from Ada’s medical knowledge database, using algorithms that have been trained on previous user queries. The net effect of this for the user is that Ada now recognizes a variety of different phrases, and links these back to specific symptoms in the database. This approach also means that Ada can now recognize new phrases after they have been entered a few times by users. It also became clear that patients often misspelled. We worked with our product team to address this issue, and Ada is now able to recognize and automatically correct a wide range of incorrectly spelt terms. Another piece of feedback received was Ada should have been made available on the primary care clinic website to facilitate at-home usage. We developed a *web embed* version deployed at scale to Sutter Health, a large health system in the United States. Several patients in the study made comments on how we could improve the treatment advice given to individuals at the end of an assessment, especially when self-care is suggested. The app now features condition-specific, high-level treatment advice for a range of minor conditions where self-care is typically appropriate.

### Future Research

Currently, the Ada symptom assessment tool is intended to be used at home. This study adds information on how patients’ intention for a primary care practice visit may change based on home use of an app. The study also provides data on the potential for symptom checkers to be used as a waiting room tool. Here, the combined ability to collect, record, and assess patients’ symptoms, and to provide advice about the most appropriate care may find a role in practice; for example, perhaps based on a fast-track app-supported doctor triage, or based on redirecting a patient to a nurse, pharmacist, or other health care practitioner within the GP practice. Such approaches will be investigated in further clinical evaluation, which will address the absolute appropriateness and safety of changes in patient intention after symptom checker use.

In addition to usability, novel digital approaches must undergo rigorous evaluation of diagnostic coverage, accuracy, and safety. In a preprint from our group (currently undergoing peer review), we evaluated the performance of 8 popular symptom checkers against one another and 7 human GP raters, as well as a *gold-standard* diagnostic suggestion using 200 clinical vignettes [31]. There was a range of coverage from the apps, with up to half of potential users being ineligible to use the symptom checker because they were too young, too old, or were pregnant; Ada offered 99.0% of users a suggested condition diagnosis. When suggesting potential diagnoses, human GPs made correct suggestions among their top 3 an average of 82.1% (SD 5.2%) of the time; the symptom checkers ranged from a low top-3 condition diagnosis accuracy of 23.5%, to Ada’s top-3 condition diagnosis accuracy of 70.5%, coming up on top of the symptom checker range and therefore closest to the performance of human GPs. In terms of safety, human GPs made a safe recommendation of what a symptom checker user should do next an average of 97.0% (SD 2.5%) of the time; Ada’s performance was identical at 97.0%.

Symptom checkers that undergo rigorous testing and certification have the potential to become useful tools to deploy alongside human medical staff to reduce diagnostic errors, prioritize sparse health resources, and improve documentation and efficiency of history taking. Diagnostic errors are all too common in our existing primary health care systems, with a systematic review commissioned by the World Health Organization suggesting around 2-3 safety incidents per 100 consultations in primary care, with many of these relating to incomplete or incorrect documentation and insufficient communication between patients and providers [3]. Another analysis from a large US population suggests a misdiagnosis rate by physicians of about 5% [22]. While software can be systematically updated, upgraded, and patched at scale, the same is not true for the existing medical system. The ideal situation would be scalable digital systems that can help the time of physicians be more appropriately allocated to the many skills that are beyond the current reach of digital technologies.

### Conclusions

Digital symptom checkers such as Ada could have a useful role to play in more appropriately directing patients to the right care in the right place at the right time.

## Appendix

Multimedia Appendix 1 Paxton Green NHS Study Survey Instrument.

## Abbreviations

GP: general practitioner
NHS: National Health Service
