# Supplementary material for: Patients’ Utilization and Perception of an Artificial Intelligence–Based Symptom Assessment and Advice Technology in a British Primary Care Waiting Room: Exploratory Pilot Study
Source: JMIR Hum Factors. 2020 Jul 10;7(3):e19713. doi: 10.2196/19713 (PMC7382011; doi:10.2196/19713)
Supplement: Multimedia Appendix 1 [file humanfactors_v7i3e19713_app1.doc]

Participant ID (for completion by researcher): ________

Age in years: _____

Sex: M / F

1. How likely are you to recommend Ada to a friend / relative?
   1. Extremely likely
   2. Likely
   3. Unlikely
   4. Extremely unlikely
2. How easy did you find Ada to use?
   1. Very easy
   2. Quite easy
   3. Quite difficult
   4. Very difficult
3. Did Ada provide helpful advice?
   1. Yes
   2. No
4. Would you use Ada again?
   1. Yes
   2. No
5. Did using Ada change your decision about what to do next?
   1. Yes - changed my mind from wanting to see a GP to self-care at home
   2. Yes - changed my mind from wanting to see a GP to visiting the pharmacy
   3. Yes - changed my mind from wanting a same day appointment to delaying my appointment for a few days
   4. Yes - changed my mind from wanting to see a GP to visiting A&E
   5. No
6. Would you have still come to the clinic if you had used Ada before coming in?
   1. Yes
   2. No
